# Creativity and schizophrenia spectrum disorders across the arts and sciences

**DOI:** 10.3389/fpsyg.2014.01145

**Published:** 2014-11-03

**Authors:** Scott Barry Kaufman, Elliot S. Paul

**Affiliations:** ^1^The Imagination InstitutePhiladelphia, PA, USA; ^2^Positive Psychology Center, University of PennsylvaniaPhiladelphia, PA, USA; ^3^Department of Philosophy, Barnard College, Columbia UniversityNew York, NY, USA

**Keywords:** mental illness, creativity, madness, schizophrenia, schizotypy, personality, schizophrenia-spectrum disorders

“*There is only one difference between a madman and me. I am not mad*.”

—*Salvador Dali*

Researchers agree that mental illness is neither necessary nor sufficient for creativity. But is there still a significant link between the two?

The oft-cited studies by Jamison (1989), Andreasen (1987), and Ludwig (1995) showing a link between mental illness and creativity have been criticized on the grounds that they involve small, highly specialized samples with weak and inconsistent methodologies and a strong dependence on subjective and anecdotal accounts (Schlesinger, 2009).

To be sure, research does show that many eminent creators—particularly in the arts—had harsh early life experiences (such as social rejection, parental loss, or physical disability) and mental, and emotional instability (Ludwig, 1995, 1998; Simonton, 1994). However, this does not mean that mental illness was a contributing factor to their eminence. There are many eminent people without mental illness or harsh early life experiences, and there is very little evidence suggesting that clinical, debilitating mental illness is conducive to productivity and innovation.

What's more, only a few of us ever reach eminence. Beghetto and Kaufman (2007) argue that we can display creativity in many different ways, from the creativity inherent in the learning process (“mini-c”), to everyday forms of creativity (“little-c”) to professional- level expertise in any creative endeavor (“Pro-c”), to eminent creativity (“Big-C”).

Engagement in everyday forms of creativity (Richards, 2007)—expressions of originality and meaningfulness in daily life—certainly do not require suffering. Quite the contrary, people who engage in everyday forms of creativity—such as making a collage, taking photographs, or publishing in a literary magazine—tend to be more open-minded, curious, persistent, positive, energetic, and intrinsically motivated by their activities (Ivcevic, 2007; Ivcevic and Mayer, 2009). Those scoring high in everyday creativity also tend to report feeling a greater sense of well-being and personal growth compared to their classmates who engage less in everyday creative behaviors.

Creating can also be therapeutic for those who are already suffering. For instance, research shows that expressive writing increases immune system functioning (Kaufman and Sexton, 2006; Kaufman and Kaufman, 2009), and the emerging field of posttraumatic growth is showing how people can turn adversity into creative growth (Tedeschi and Calhoun, 2004; Forgeard, 2013).

That said, there is a grain of truth to the notion that creativity and mental illness are related, but the truth is much more nuanced—and we think interesting—than the more romanticized notions of the link.

To see the matter clearly, we need to take a step back and consider what we mean by “creativity” and “mental illness” in the first place. While there are many forms of mental illness, this paper focuses on schizophrenia, a mental disorder characterized by a severe disconnect from reality, including a tendency to experience thoughts that are divergent, disorganized, and delusional. One aspect of creativity is obviously novelty or originality. Schizophrenic thoughts are more likely to be unique or new. So, by its very nature, schizophrenia disposes one toward satisfying one requirement for creative thought: namely originality.

Originality is not sufficient for creativity, however, for as Kant (2000) observed long ago, “there can be original nonsense,” as in the word salad of a schizophrenic patient. For a product to be creative it must not only be new but also useful, effective, or valuable in some way (Sternberg and Lubart, 1999; Gaut, 2010; Klausen, 2010). A highly original product might be deemed a symptom of mental illness or an expression of creativity depending on whether or not it is useful. Creativity is maximized when both novelty and utility are simultaneously maximized.

These two features—novelty and utility—respectively depend on two cognitive functions: the *generation* of ideas popping up in conscious thought, and the *selection* of ideas to be explored, developed, and ultimately expressed or realized in the form of an observable product (cf. Finke et al., 1992). These two cognitive functions map nicely onto the Blind Variation and Selective Retention (BVSR) model of creativity (Campbell, 1960; Simonton, 2011; Jung et al., 2013; Jung, 2014).

The more productively one *generates* ideas (regardless of the extent to which the utilities are initially known), the more likely some of them will be new. The more effectively one *selects* and develops particular ideas, the more likely some of them will result in something useful. Being creative is similar to mental illness in that it involves a heightened capacity and inclination to produce a large quantity of ideas and associations. What distinguishes the creative person is that she is better able to manage the flood of ideas, selecting the useful ones and developing them effectively while discarding the others. This chimes with the oft-quoted remark of scientist, peace activist, and two-time Nobel laureate Linus Pauling: “The way to get good ideas is to get lots of ideas and throw the bad ones away.”

Indeed, recent research suggests a link between milder forms of schizophrenia and creativity. In a recent report based on a 40-year study of roughly 1.2 million Swedish people, Kyaga et al. (2013) found that those in scientific and artistic occupations were *not* more likely to suffer from psychiatric disorders, with the exception of bipolar disorders. So full-blown mental illness did not increase the probability of entering a creative profession (even the exception, bipolar disorder, showed only a small effect of 8%).

What was striking, however, was that the *siblings* of patients with autism and the *first-degree relatives* of patients with schizophrenia were significantly overrepresented in creative professions. Could it be that the relatives inherited a watered-down version of the mental illness conducive to creativity while avoiding the aspects that are debilitating?

Research shows that psychologically healthy biological relatives of people with schizophrenia have unusually creative jobs and hobbies and tend to show higher levels of schizotypal personality traits compared to the general population (Karlsson, 1970; Kinney et al., 2001). Schizotypy consists of a constellation of personality traits that are evident to some degree in everyone.

Schizotypal traits can be broken down into two types. “Positive” schizotypy includes unusual perceptual experiences, thin mental boundaries between self and other, impulsive nonconformity, and magical beliefs. “Negative” schizotypal traits include cognitive disorganization and physical and social anhedonia (difficulty experiencing pleasure from social interactions and activities that are enjoyable for most people). Nettle (2006) found that people with schizotypy typically resemble schizophrenia patients much more along the positive schizotypal dimensions (such as unusual experiences) compared to the negative schizotypal dimensions (such as lack of affect and volition).

This has important implications for creativity. Batey and Furnham (2008) found that the unusual experiences and impulsive nonconformity dimensions of schizotypy, but not the cognitive disorganization dimension, were significantly related to self-ratings of creativity, a creative personality (measured by a checklist of adjectives such as “confident,” “individualistic,” “insightful,” “wide interests,” “original,” “reflective,” “resourceful,” “unconventional,” and “sexy”), and everyday creative achievement among thirty-four activities (“written a short story,” “produced your own website,” “composed a piece of music,” and so forth).

Recent neuroscience findings further support the link between schizotypy and creative cognition. Takeuchi et al. (2011) investigated the functional brain characteristics of participants while they engaged in a difficult working memory task. Importantly, none of their subjects had a history of neurological or psychiatric illness, and all had intact working memory abilities. Participants were asked to display their creativity in a number of ways: generating unique ways of using typical objects, imagining desirable functions for ordinary objects and imagining the consequences of “unimaginable things” happening.

The researchers found that the more creative the participant, the more they had difficulty suppressing the precuneus while engaging in an effortful working memory task. The precuneus is the area of the Default Network (Buckner et al., 2008; Jung et al., 2013; Andrews-Hanna et al., 2014) that typically displays the highest levels of activation during rest (when a person is not focusing on an external task). The precuneus has been linked to self-consciousness, self-related mental representations, and the retrieval of personal memories (Cavanna and Trimble, 2006). How is this conducive to creativity? According to the researchers, “Such an inability to suppress seemingly unnecessary cognitive activity may actually help creative subjects in associating two ideas represented in different networks.”

Whitfield-Gabrieli et al. (2009) found a similar inability to deactivate the precuneus among schizophrenic individuals and their relatives. Which raises the intriguing question: what happens if we directly compare the brains of creative people against the brains of people with schizotypy?

A recent study by Fink et al. (2014) sheds some light on this question. Consistent with earlier research, they found an association between the ability to come up with original ideas and the inability to suppress activation of the precuneus during creative thinking. As the researchers note, these findings are consistent with the idea that more creative people include more events/stimuli in their mental processes than less creative people. But crucially, they found that those scoring high in schizotypy showed a similar pattern of brain activations during creative thinking as the highly creative participants. This supports the idea that overlapping mental processes are implicated in both creativity *and* psychosis proneness.

Therefore, it seems that the key to creative cognition is opening up the flood gates and letting in as much information as possible. Because you never know: sometimes the most bizarre associations can turn into the most productively creative ideas. This idea is consistent with recent research on latent inhibition (Kaufman, 2009). Latent inhibition is a filtering mechanism that we share with other animals and it is tied to the neurotransmitter dopamine (Lubow and Weiner, 2010). A reduced latent inhibition allows us to treat something as novel, no matter how many times we've seen it before and tagged it as irrelevant.

Prior research shows a link between reduced latent inhibition and acute-phase schizophrenia (Baruch et al., 1988a,b; Lubow et al., 1992). But more recent research also shows a link to creativity. Carson et al. (2003) found that the most eminent creative achievers among a sample of Harvard undergrads were seven times more likely to have reduced latent inhibition. As Carson (2011) points out in her “Shared Vulnerability Model,” mental processes such as reduced latent inhibition, preference for novelty, and hyperconnectivity can “enlarge the range and depth of stimuli available in conscious awareness to be manipulated and combined to form novel and original ideas” (p. 144). Extreme levels of these factors make one vulnerable to severely disordered thinking. But they can be mitigated and channeled productively if one has *protective factors*, such as enhanced fluid reasoning, working memory, cognitive inhibition, and cognitive flexibility (Kuszewski, 2009; Carson, 2011).

Another protective factor may lie within the openness to experience domain, a broad personality domain reflecting the tendency toward cognitive exploration (DeYoung, 2014). Peterson and Carson (2000) and Peterson et al. (2002) found that students with reduced latent inhibition scored higher in openness to experience. But while openness to experience is consistently associated with creativity (see Kaufman, 2013, Kaufman et al., submitted), this personality domain can also be meaningfully separated into distinct (but correlated) subtraits of *Openness to Experience* and *Intellect* (DeYoung et al., 2007; DeYoung, 2014).

*Openness to Experience* reflects cognitive engagement with sensory and perceptual information, whereas *Intellect* reflects cognitive engagement with abstract and semantic information, primarily through reasoning. While Intellect is associated with IQ, executive functioning, and intellectual engagement, Openness is associated with fantasy-proneness, schizotypy, absorption, delusional ideation, and the tendency to make connections and see patterns that don't actually exist (DeYoung et al., 2012; Kaufman, 2013; Menon et al., 2013; Chmielewski et al., 2014). Indeed, Intellect is negatively associated with positive schizotypy and delusional ideation (Menon et al., 2013).

Therefore, the combination of Openness and Intellect may be crucial to maintaining high levels of creative production (DeYoung et al., 2012). The proper balance most likely differs by domain. There is emerging evidence across diverse samples, ages, and occupations that Openness is associated with creative achievement in the arts, whereas Intellect is associated with creative achievement in the sciences (Kaufman, 2013; Kaufman et al., submitted). This is consistent with research suggesting that schizotypy is associated with verbal and artistic creativity (Del Giudice et al., 2010; Beaussart et al., 2012) whereas the autism spectrum is associated with technical-scientific interests and careers (Baron-Cohen et al., 2001; Crespi and Badcock, 2008).

Nevertheless, recent research suggests that creative cognition draws on both the executive functioning that is tied to Intellect and the associative divergence that comes with Openness (Nusbaum and Silvia, 2011; Beaty et al., 2014; Benedek et al., 2014; Jung, 2014). Being susceptible to schizophrenia spectrum disorders may enhance Openness, increasing the likelihood of ideas that are original. To develop ideas that are creative, however, one also needs protective intellectual factors (and autistic-like traits) to steer the chaotic storm.

## Author note

Portions of this article were taken from the blog post “The Real Link Between Mental Illness and Creativity” (http://blogs.scientificamerican.com/beautiful-minds/2013/10/03/the-real-link-between-creativity-and-mental-illness/), published by the first author at Scientific American on October 3, 2013.

### Conflict of interest statement

The authors declare that the research was conducted in the absence of any commercial or financial relationships that could be construed as a potential conflict of interest.

## References

[B1] Andreasen N. C. (1987). Creativity and mental illness: prevalence rates in writers and their first-degree relatives. Am. J. Psychiatry 144, 1288–1292. 3499088 10.1176/ajp.144.10.1288

[B2] Andrews-Hanna J. R. Smallwood J. Spreng R. N. (2014). The default network and self-generated thought: component processes, dynamic control, and clinical relevance. Ann. N.Y. Acad. Sci. 1316, 29–52. 10.1111/nyas.12360 24502540 PMC4039623

[B3] Baron-Cohen S. Wheelwright S. Skinner R. Martin J. Clubley E. (2001). The Autism-Spectrum Quotient (AQ): evidence from Asperger syndrome/high-functioning autism, males and females, scientists and mathematicians. J. Autism Dev. Dis. 31, 5–17. 10.1023/A:1005653411471 11439754

[B3a] Baruch I. Hemsley D. R. Gray J. A. (1988a). Differential performance of acute and chronic schizophrenics in a latent inhibition task. J. Nerv. Ment. Dis. 176, 598–606. 10.1097/00005053-198810000-00004 2903219

[B3b] Baruch I. Hemsley D. R. Gray J. A. (1988b). Latent inhibition and “psychotic proneness” in normal subjects. Pers. Individ. Dif. 9, 777–783. 10.1016/0191-8869(88)90067-08467369

[B4] Batey M. Furnham A. (2008). The relationship between measures of creativity and schizotypy. Pers. Individ. Dif. 45, 816–821. 10.1016/j.paid.2008.08.014

[B5] Beaty R. E. Silvia P. J. Nusbaum E. C. Jauk E. Benedek M. (2014). The roles of associative and executive processes in creative cognition. Mem. Cogn. 42, 1186–1197. 10.3758/s13421-014-0428-8 24898118

[B6] Beaussart M. L. Kaufman S. B. Kaufman J. C. (2012). Creative activity, personality, mental illness, and short-term mating success. J. Creat. Behav. 46, 151–167. 10.1002/jocb.11

[B7] Beghetto R. A. Kaufman J. C. (2007). Toward a broader conception of creativity: a case for “mini-c” creativity. Psychol. Aesthetics Creativity Arts 1:73. 10.1037/1931-3896.1.2.73

[B8] Benedek M. Jauk E. Sommer M. Arendasy M. Neubauer A. C. (2014). Intelligence, creativity, and cognitive control: the common and differential involvement of executive functions in intelligence and creativity. Intelligence 46, 73–83. 10.1016/j.intell.2014.05.007 25278640 PMC4175011

[B9] Buckner R. L. Andrews-Hanna J. R. Schacter D. L. (2008). The brain's default network. Ann. N.Y. Acad. Sci. 1124, 1–38. 10.1196/annals.1440.011 18400922

[B10] Campbell D. T. (1960). Blind variation and selective retention in creative thought as in other knowledge processes. Psychol. Rev. 67, 380–400. 10.1037/h0040373 13690223

[B11] Carson S. H. (2011). Creativity and psychopathology: a shared vulnerability model. Can. J. Psychiatry 56, 144–153. 21443821 10.1177/070674371105600304

[B12] Carson S. H. Peterson J. B. Higgins D. M. (2003). Decreased latent inhibition is associated with increased creative achievement in high-functioning individuals. J. Pers. Soc. Psychol. 85:499. 10.1037/0022-3514.85.3.499 14498785

[B13] Cavanna A. E. Trimble M. R. (2006). The precuneus: a review of its functional anatomy and behavioural correlates. Brain 129, 564–583. 10.1093/brain/awl004 16399806

[B14] Chmielewski M. Bagby M. Markon K. Ring A. J. Ryder A. G. (2014). Openness to experience, intellect, schizotypal personality disorder, and psychoticism: resolving the controversy. J. Pers. Disord. 28, 483–499. 10.1521/pedi_2014_28_128 24511900

[B15] Crespi B. Badcock C. (2008). Psychosis and autism as diametrical disorders of the social brain. Behav. Brain Sci. 31, 241–320. 10.1017/S0140525X08004214 18578904

[B15a] Del Giudice M. Angeleri R. Brizio A. Elena M. R. (2010). The evolution of autistic-like and schizotypal traits: a sexual selection hypothesis. Front. Psychol. 1:41. 10.3389/fpsyg.2010.00041 21833210 PMC3153759

[B16] DeYoung C. G. (2014). Openness/Intellect: a dimension of personality reflecting cognitive exploration, in APA Handbook of Personality and Social Psychology, Vol. 3, Personality Processes and Individual Differences, eds Cooper M. L. Larsen R. J. (Washington, DC: American Psychological Association).

[B17] DeYoung C. G. Grazioplene R. G. Peterson J. B. (2012). From madness to genius: the Openness/Intellect trait domain as a paradoxical simplex. J. Res. Pers. 46, 63–78. 10.1016/j.jrp.2011.12.003

[B16a] DeYoung C. G. Quilty L. C. Peterson J. (2007). Between facets and domains: 10 aspects of the Big Five. J. Pers. Soc. Psychol. 93, 880–896. 10.1037/0022-3514.93.5.880 17983306

[B18] Fink A. Weber B. Koschutnig K. Benedek M. Reishofer G. Ebner F.. (2014). Creativity and schizotypy from the neuroscience perspective. Cogn. Affect. Behav. Neurosci. 14, 378–387. 10.3758/s13415-013-0210-6 24022793

[B19] Finke R. A. Ward T. B. Smith S. M. (1992). Creative Cognition: Theory, Research, and Applications. Cambridge, MA: Bradford

[B20] Forgeard M. J. C. (2013). Perceiving benefits after adversity: the relationship between self-reported posttraumatic growth and creativity. Psychol. Aesthetics Creativity Arts 7, 245–264. 10.1037/a0031223

[B21] Gaut B. (2010). The philosophy of creativity. Philos. Compass 5, 1034–1046. 10.1111/j.1747-9991.2010.00351.x

[B22] Ivcevic Z. (2007). Artistic and everyday creativity: an act-frequency approach. J. Creat. Behav. 41, 271–290. 10.1002/j.2162-6057.2007.tb01074.x

[B23] Ivcevic Z. Mayer J. D. (2009). Mapping dimensions of creativity in the life-space. Creativity Res. J. 21, 152–165. 10.1080/10400410902855259

[B24] Jamison K. R. (1989). Mood disorders and patterns of creativity in British writers and artists. Psychiatry 52, 125–134. 2734415 10.1080/00332747.1989.11024436

[B25] Jung R. E. (2014). Evolution, creativity, intelligence, and madness: “Here Be Dragons.” Front. Psychol. 5:784. 10.3389/fpsyg.2014.00784 25101040 PMC4107956

[B26] Jung R. E. Mead B. S. Carrasco J. Flores R. A. (2013). The structure of creative cognition in the human brain. Front. Hum. Neurosci. 7:330. 10.3389/fnhum.2013.00330 23847503 PMC3703539

[B27] Kant I. (2000). Critique of the Power of Judgment, ed Guyer Paul New York, NY: Cambridge University Press

[B27a] Karlsson J. L. (1970). Genetic association of giftedness and creativity with schizophrenia. Hereditas 66, 177–182. 5525825 10.1111/j.1601-5223.1970.tb02343.x

[B28] Kaufman J. C. Sexton J. D. (2006). Why doesn't the writing cure help poets? Rev. Gen. Psychol. 10, 268–282. 10.1037/1089-2680.10.3.268

[B29] Kaufman S. B. (2009). Faith in intuition is associated with decreased latent inhibition in a sample of high-achieving adolescents. Psychol. Aesthetics Creativity Arts 1, 28–34. 10.1037/a0014822

[B30] Kaufman S. B. (2013). Opening up openness to experience: a four-factor model and relations to creative achievement in the arts and sciences. J. Creat. Behav. 47, 233–255. 10.1002/jocb.33

[B31] Kaufman S. B. Kaufman J. C. (2009). The Psychology of Creative Writing. New York, NY: Cambridge University Press

[B33] Kinney D. K. Richards R. Lowing P. A. LeBlanc D. Zimbalist M. E. Harlan P. (2001). Creativity in offspring of schizophrenic and control parents: an adoption study. Creativity Res. J. 13, 17–25. 10.1207/S15326934CRJ1301_3

[B34] Klausen S. H. (2010). The notion of creativity revisited: a philosophical perspective on creativity research. Creativity Res. J. 22, 347–360. 10.1080/10400419.2010.523390

[B35] Kuszewski A. (2009). The Genetics of Creativity: A Serendipitous Assemblage of Madness. Metodo Working Papers no. 58, Bogotá

[B36] Kyaga S. Landén M. Boman M. Hultman C. M. Långström N. Lichtenstein P. L. (2013). Mental illness, suicide and creativity: 40-year prospective total population study. J. Psychiatr. Res. 47, 83–90. 10.1016/j.jpsychires.2012.09.010 23063328

[B36a] Lubow R. E. Ingberg-Sachs Y. Zalstein-Orda N. Gewirtz J. C. (1992). Latent inhibition in low and high “psychotic-prone” normal subjects. Pers. Individ. Dif. 15, 563–572. 10.1016/0191-8869(92)90197-W8467369

[B37] Lubow R. Weiner I. (eds.). (2010). Latent Inhibition: Cognition, Neuroscience and Applications to Schizophrenia. New York, NY: Cambridge University Press. 10.1017/CBO9780511730184

[B38] Ludwig A. M. (1995). The Price of Greatness: Resolving the Creativity and Madness Controversy. New York, NY: Guilford Press

[B39] Ludwig A. M. (1998). Method and madness in the arts and sciences. Creativity Res. J. 11, 93–101. 10.1207/s15326934crj1102_1

[B40] Menon M. Quilty L. C. Zawadzki J. A. Woodward T. S. Sokolowski H. M. Boon H. S.. (2013). The role of cognitive biases and personality variables in subclinical delusional ideation. Cogn. Neuropsychiatry 18, 208–218. 10.1080/13546805.2012.692873 22758619

[B41] Nettle D. (2006). Schizotypy and mental health amongst poets, visual artists, and mathematicians. J. Res. Pers. 40, 876–890. 10.1016/j.jrp.2005.09.004

[B42] Nusbaum E. C. Silvia P. J. (2011). Are intelligence and creativity really so different? Fluid intelligence, executive processes, and strategy use in divergent thinking. Intelligence 39, 36–45. 10.1016/j.intell.2010.11.002

[B43] Peterson J. Carson S. (2000). Latent inhibition and openness to experience in a high-achieving student population. Pers. Individ. Dif. 28, 323–332. 10.1016/S0191-8869(99)00101-4

[B44] Peterson J. Smith K. W. Carson S. (2002). Openness and extraversion are associated with reduced latent inhibition: replication and commentary. Pers. Individ. Dif. 33, 1137–1147. 10.1016/S0191-8869(02)00004-1

[B45] Richards R. E. (2007). Everyday Creativity and New Views of Human Nature: Psychological, Social, and Spiritual Perspectives. Washington, DC: American Psychological Association

[B46] Schlesinger J. (2009). Creative mythconceptions: a closer look at the evidence for the “mad genius” hypothesis. Psychol. Aesthetics Creativity Arts 3, 62. 10.1037/a0013975

[B47] Simonton D. K. (1994). Greatness: Who Makes History and Why. New York, NY: Guilford Press

[B48] Simonton D. K. (2011). Creativity and discovery as blind variation: Campbell's 1960 BVSR model after the half-century mark. Rev. Gen. Psychol. 15, 158–174. 10.1037/a0022912

[B49] Sternberg R. J. Lubart T. I. (1999). The concept of creativity: prospects and paradigms, in Handbook of Creativity, ed Sternberg R. J. (Cambridge, UK: Cambridge University Press), 3–15

[B50] Takeuchi H. Taki Y. Hashizume H. Sassa Y. Nagase T. Nouchi R.. (2011). Failing to deactivate: the association between brain activity during a working memory task and creativity. Neuroimage 55, 681–687. 10.1016/j.neuroimage.2010.11.052 21111830

[B51] Tedeschi R. G. Calhoun L. G. (2004). Posttraumatic Growth: conceptual foundations and empirical evidence. Psychol. Inq. 15, 1–18. 10.1207/s15327965pli1501_01

[B53] Whitfield-Gabrieli S. Thermenos H. W. Milanovic S. Tsuang M. T. Faraone S. V. McCarley R. W.. (2009). Hyperactivity and hyperconnectivity of the default network in schizophrenia and in first-degree relatives of persons with schizophrenia. Proc. Natl. Acad. Sci. U.S.A. 106, 1279–1284. 10.1073/pnas.0809141106 19164577 PMC2633557

